# Extracts from New Zealand *Undaria pinnatifida* Containing Fucoxanthin as Potential Functional Biomaterials against Cancer *in Vitro*

**DOI:** 10.3390/jfb5020029

**Published:** 2014-03-31

**Authors:** Sheng Kelvin Wang, Yan Li, William Lindsey White, Jun Lu

**Affiliations:** 1 School of Applied Sciences, Faculty of Health and Environment Sciences, Auckland University of Technology, Auckland 1142, New Zealand; E-Mails: kelwang@aut.ac.nz (S.K.W.); yan.li@aut.ac.nz (Y.L.); lindsey.white@aut.ac.nz (W.L.W.); 2 Institute of Biomedical Technology, Auckland University of Technology, Auckland 1142, New Zealand; 3 School of Interprofessional Health Studies, Faculty of Health and Environment Sciences, Auckland University of Technology, Auckland 1142, New Zealand

**Keywords:** fucoxanthin, *Undaria pinnatifida*, anti-cancer, New Zealand seaweed, extract

## Abstract

This study tested extracts from New Zealand seaweed *Undaria pinnatifida* containing fucoxanthin, in parallel with pure fucoxanthin, in nine human cancer cell lines, for anticancer activity. Growth inhibition effects of extracts from *Undaria pinnatifida* were found in all types of cancer cell lines in dose- and time- dependent manners. Cytotoxicity of fucoxanthin in three human non-cancer cell lines was also tested. Compared with pure fucoxanthin, our extracts containing low level of fucoxanthin were found to be more effective in inhibiting the growth of lung carcinoma, colon adenocarcinoma and neuroblastoma. Our results suggest that fucoxanthin is a functional biomaterial that may be used as a chemopreventive phytochemical or in combination chemotherapy. Furthermore, we show for the first time that some unknown compounds with potential selective anti-cancer effects may exist in extracts of New Zealand *Undaria pinnatifida*, and New Zealand *Undaria pinnatifida* could be used as a source for either functional biomaterial extraction or production of functional food.

## 1. Introduction

The ocean represents an abundant resource of novel compounds with great potential for pharmaceutical, nutritional supplements, cosmetics, agrichemicals and enzymes [1]. The brown algal genus *Undaria* (*Phaeophyceae*, Order *Laminariales*, Family *Alariaceae*) has been an important dietary component since at least sixth century in most Asian countries. *Undaria pinnatifida* (*U. pinnatifida*) was accidently introduced into New Zealand (NZ) where it was first discovered in the Wellington Harbor in 1987 [2]. It is listed as an “unwanted organism” by Biosecurity NZ. As a result, no one could collect it and little research has been done on it for more than two decades, and the seaweed spread throughout the east coast of NZ primarily on ropes associated with mussel farms. In May 2010, recognizing that there is no chance to eradicate this seaweed, the Biosecurity NZ allowed for permits to be issued for the collection of *U. pinnatifida* from artificial structures (e.g., mussel farms) and for it to be farmed in areas where it is already prolific. *Undaria pinnatifida* is a source of biologically active phytochemicals, including a wide range of components such as carotenoids, phycobilins, fatty acids, polysaccharides, vitamins, sterols, tocopherol, and phycocyanins [3]. The major components in *U. pinnatifida* such as fucoxanthin and fucoidan have been reported to induce apoptosis in various tumor cells [4,5,6,7,8].

**Figure 1:**
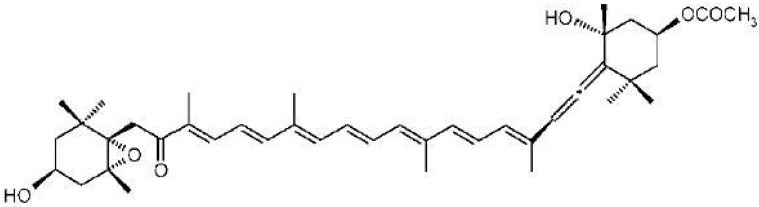
The structure of fucoxanthin.

Fucoxanthin is an abundant marine xanthophyll, and it is the most important pigment in generating the brown color in brown seaweeds [9]. The structure of fucoxanthin (Figure 1) contains an unusual allelic bond and some oxygenic functional groups such as epoxy, hydroxyl, carbonyl and carbonyl moieties. It is a type of carotenoid and possesses anti-oxidative and cancer cell growth inhibition effects. Free radicals and oxidative stress are involved in the pathogenesis of cancer disease [9]. *In vitro* studies have demonstrated that fucoxanthin induces apoptosis and enhances growth inhibition effects on several cancer cell lines including human promyelocytic leukemia HL-60 cell line [10], human colon carcinoma including Caco-2, WiDr, HT-29 and DLD-1 [5], human lung bronchopulmonary and epithelial cancer cells NSCLC-N6 and A549 [11], human prostate cancer cell lines such as PC-3, DU 145, and LNCaP [12], human hepatocellular cancer cell Hep G2 [13], human gastric adenocarcinoma MGC-803 [14], and human neuroblastoma GOTO cells [15].

In order to take full advantage of the benefit of *U. pinnatifida* growing in NZ, it is important and necessary to fully understand its possible chemical composition and potential use, because even the same species growing in different environment may produce different functional biomaterial composition, as well as bioactivity. In this study, we investigated the cancer cell growth inhibition of extracts, mainly containing fucoxanthin, from *U. pinnatifida* grown in Marlborough Sounds, South Island of NZ.

## 2. Results and Discussion

### 2.1. HPLC Quantification of Fucoxanthin in U. pinnatifida Extracts

**Figure 2:**
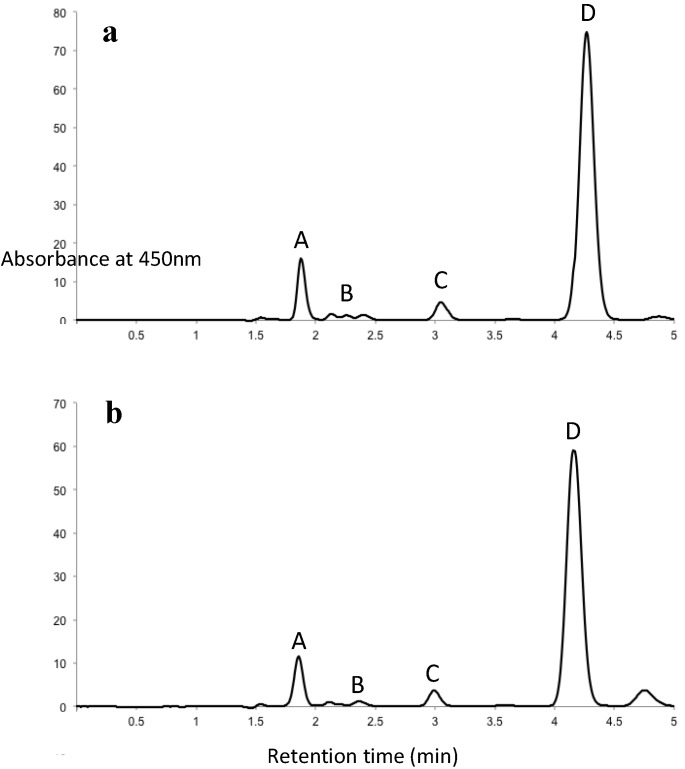
HPLC chromatograms of (**a**) the 1st NZ *U. pinnatifida* extract; (**b**) the 2nd NZ *U. pinnatifida* extract. Peaks in chromatograms: (A) *trans*-fucoxanthin; (B) *cis*-fucoxanthin; (C) unidentified peak; (D) canthaxanthin (internal standard).

Fucoxanthin was extracted from the blade (the main part of the plant) and sporophyll (the reproductive structure that sits at the bottom of the blade near the plant’s attachment to the substrate) of *U. pinnatifida*, and the HPLC chromatograms of the first and second fucoxanthin extracts are shown in Figure 2. Previous data from literature showed that *trans-*fucoxanthin was the major isomer (~88%) found in fresh brown seaweeds, and a small amount was 13-*cis* and 13’-*cis* isomers (~9%) [16]. In this study, fucoxanthin was detected at 450 nm wavelength. *Trans*-fucoxanthin peak appeared at the retention time of around 1.8 min. The two peaks ascribed as the 13-*cis* and 13’-*cis* isomers of fucoxanthin were detected at retention times of 2.2 min and 2.4 min, respectively. The peak detected at 4.3 min was the internal standard canthaxanthin. An unidentified peak was detected at a retention time of 3.0 min in both blade and sporophyll. This peak was not purified and investigated further in this study. Based on HPLC quantification, the crude, first and second extract contained 0.2%, 43.5% and 60.8% fucoxanthin, respectively. The ratio of Peak C’s area under the curve against that of the internal standard (AUC_peakC_/AUC_IS_) increased with decreased fucoxanthin content in extracts. The crude contained the highest AUC_peakC_/AUC_IS_ and the 2nd extract contained the lowest AUC_peakC_/AUC_IS_.

### 2.2. Pure Fucoxanthin on Cancer Cell Growth

**Figure 3:**
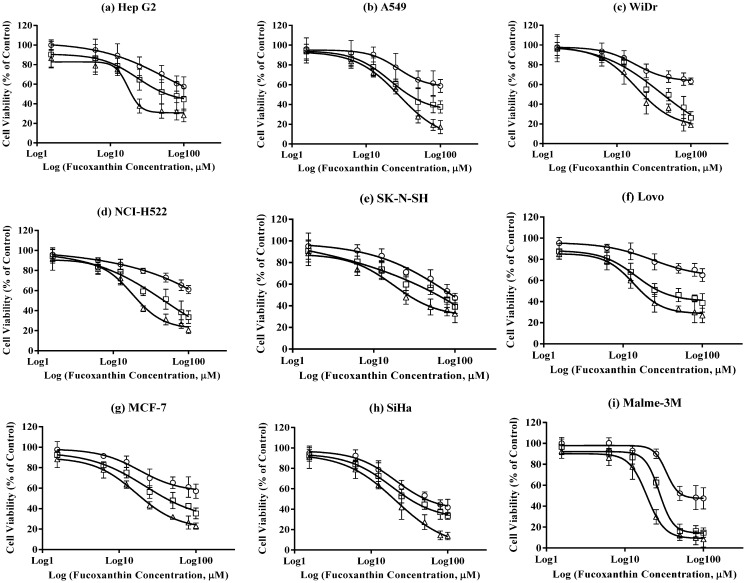
Inhibitory effects of pure fucoxanthin standard. All nine cancer cell lines were treated with 1.5625, 6.25, 12.5, 25, 50, 80, 100 μM fucoxanthin. Inhibitory effects were determined after treatment at 24 h (○), 48 h (□) and 72 h (Δ). Data are expressed as mean ± standard errors (n = 6). (**a**) In Hep G2 cell line; (**b**) in A549 cell line; (**c**) in WiDr cell line; (**d**) in NCI-H522 cell line; (**e**) in SK-N-SH cell line; (**f**) in Lovo cell line; (**g**) in MCF-7 cell line; (**h**) in SiHa cell line; (**i**) in Malme-3M cell line.

All nine cancer cell lines were treated with pure fucoxanthin (Sigma) at concentrations of 1.5625, 6.25, 12.5, 25, 50, 80, 100 µM for 24, 48 and 72 h, respectively (Figure 3). There was significant difference between high and low concentrations and between 24, 48 and 72 h treatments (*p* < 0.05). This suggests fucoxanthin’s cancer cell growth inhibition effect is time- and dose-dependent. 

For 24-h incubation, SK-N-SH (Figure 3e), SiHa (Figure 3h), and Malme-3M cell lines (Figure 3i) showed higher sensitivity to fucoxanthin treatment. For human malignant melanoma cell line Malme-3M, cell viability could be significantly decreased starting from 50 µM treatment within 24 h. This provides the foundation for further investigation on whether fucoxanthin can be used topically for the treatment of melanoma or in combination therapy to assist other melanoma treatments. 

In 48-h incubations, cell viability in most cancer cell lines decreased in the presence of low fucoxanthin concentration. After treatment with pure fucoxanthin standard at 100 µM for 72 h, cell viability in all tested cancer cell lines decreased to approximately 20% compared with untreated cell cultures.

The IC_50_ values for growth inhibition effects of pure fucoxanthin standard on all cancer cell lines are shown in Table 1. The first and the most obvious feature was that the lowest IC_50_ values were found in human malignant melanoma Malme-3M and human cervix squamous carcinoma SiHa cells. IC_50_ values for Lovo, MCF-7, NCI-H522, and A549 cell lines were higher than those of Malme-3M and SiHa, but lower than other types of cell lines (*p* < 0.05). Human neuroblastoma SK-N-SH seemed to be less sensitive to pure fucoxanthin treatment.

**Table 1:** Inhibition effects IC_50_ comparison from pure fucoxanthin standard treatment in all nine types of cancer cell lines for 48 and 72 h. The *p*-values were comparison of IC_50_ with Malme-3M and SiHa cell lines. Data are expressed as mean ± standard errors (n = 6).

Cancer Cell Lines	IC_50_ after Fucoxanthin Treatment for 48 h	IC_50_ after Fucoxanthin Treatment for 72 h	*p*-values
Hep G2	58.89 ± 1.23 μM	25.9 ± 1.69 μM	*p* < 0.05
A549	44.7± 2.26 μM	25.57 ± 1.07 μM	*p* < 0.05
WiDr	42.26 ± 1.25 μM	25.03 ± 1.63 μM	*p* < 0.05
NCI-H522	46.11 ± 1.07 μM	24.46 ± 1.58 μM	*p* < 0.05
SK-N-SH	52.69 ± 1.07 μM	31.21 ± 2.62 μM	*p* < 0.01
Lovo	39.62 ± 0.94 μM	21.83 ± 1.17 μM	*p* < 0.05
MCF-7	43.96 ± 2.61 μM	22.48 ± 1.26 μM	*p* < 0.05
SiHa	37.6± 1.49 μM	18.9 ± 2.16 μM	–
Malme-3M	27.96 ± 1.36 μM	17.33 ± 2.65 μM	–

There are several mechanisms of action that have been proposed for fucoxanthin’s cancer cell growth inhibition effect. First, it could induce cell apoptosis reducing the proliferation of human breast adenocarcinoma MCF-7 [17,18,19]. Second, it might contribute to morphological change, triggering the terminal differentiation of human lung carcinoma A549 *in vitro* [11]. Third, in human hepatocellular carcinoma Hep G2 and colon adenocarcinoma WiDr, it could arrest the G0/G1 phase of the cell cycle and direct inhibition of the DNA replication step. Currently, there is no mechanism of action proposed for fucoxanthin’s cancer cell growth inhibition effect on melanoma and cervix squamous carcinoma. The results from our study suggest that those two cancer cell lines are very sensitive to the growth inhibition effect of pure fucoxanthin. Thus, further study of mechanism of fucoxanthin’s action in those types of cancer is warranted, especially in melanoma, because fucoxanthin may be topically used, therefore avoiding the complicated issues of drug absorption, metabolism and distribution.

### 2.3. Analysis of Fucoxanthin Treatment in Human Non-Cancer Cells

The most sensitivity to growth inhibition effects of fucoxanthin was found in HUVEC cell lines. HDFb and HEK293 were more tolerant to the treatment of fucoxanthin (see IC_50_ summary in Table 2). The IC_50_ appeared to be lower than in most cancer cell lines. However, at concentration lower than IC_50_, fucoxanthin has less effect on the growth of human non-cancer cell lines. For example, in the control study, fucoxanthin at 1.56 μM could even promote the growth of human non-cancer cells. For HEK293, fucoxanthin still had no growth inhibition effect on cells at 6.25 μM during 24- and 48-h treatment. The control study suggests that fucoxanthin at low concentration could exert selective cancer cell growth inhibition effect on cancer cells but not on normal human cells.

**Table 2:** Comparison of inhibitory effects IC_50_ values in all nine cancer cell lines and three non-cancer cell lines between treatment of pure fucoxanthin and three New Zealand *U. pinnatifida* extracts including crude (contains 0.2% fucoxanthin), first (contains 43.5% fucoxanthin) and second (contains 60.8% fucoxanthin) extracts. Data show IC50 (μM) after 48-h treatment/72-h treatment, and are expressed as mean ± standard error (n = 6).

Cell lines	Pure fucoxanthin	Second extract	First extract	Crude extract
*Cancer cell line*				
Hep G2	(58.89 ± 1.23)/ (25.90 ± 1.69)	(48.03 ± 2.08)/ (30.85 ± 0.46)	(43.04 ± 1.96)/ (25.37 ± 1.07)	(39.6 ± 2.00)/ (25.50 ± 1.40)
A549	(44.70 ± 2.26)/ (25.57 ± 1.07)	(49.03 ± 1.02 )/ (28.89 ± 2.67)	(48.88 ± 0.91)/ (28.89 ± 2.67)	(47.68 ± 0.26)/ (21.51 ± 0.54)
NCI-H522	(46.11 ± 1.07)/ (24.46 ± 1.58)	(50.89 ± 2.27)/ (25.87 ± 2.07)	(49.61 ± 2.01)/ (26.58 ± 1.53)	(31.00 ^a^ ± 1.41)/ (14.58 ^a^ ± 0.96)
WiDr	(42.26 ± 1.25)/ (25.03 ± 1.63)	(42.33 ± 1.53)/ (23.75 ± 2.63)	(47.11 ± 1.07)/ (20.12 ± 2.95)	(24.06 ^a^ ± 0.57)/ (15.83 ^a^ ± 0.44)
Lovo	(39.62 ± 0.94)/ (21.83 ± 1.17)	(40.40 ± 2.30)/ (17.39 ± 2.00)	(30.46 ± 0.59)/ (12.67 ± 0.16)	(14.18 ^a^ ± 0.52)/ (8.84 ^a^ ± 0.38)
SK-N-SH	(52.69 ± 2.07)/ (31.21 ± 1.62)	(32.92 ^a^ ± 2.28)/ (25.51 ^a^ ± 4.02)	(30.79 ^a^ ± 2.32)/ (17.57 ^a^ ± 1.49)	(30.42 ^a^ ± 2.41)/ (20.01 ^a^ ± 2.42)
MCF-7	(43.96 ± 2.61)/ (22.48 ± 1.26)	(37.59 ± 2.29)/ (22.93 ± 2.83)	(37.38 ± 2.27)/ (27.39 ± 1.83	35.63 ± 1.65)/ (25.12 ±3.46)
SiHa	(37.76 ± 1.49)/ (18.9 ± 2.16)	(29.98 ± 1.52)/ (18.84 ± 1.42)	(32.8 ± 2.53)/ (21.34 ± 1.17)	(30.86 ± 2.73)/ (18.9 ± 2.16)
Malme-3M	(27.96 ± 1.36)/ (17.33 ± 2.65)	(49.76 ± 2.30)/ (35.09 ± 2.03)	(44.12 ± 1.86)/ (29.8 ± 1.36)	(33.9 ± 2.99)/ (25.13 ± 2.72)
*Non-cancer cell line*				
HDFB	(32.23 ± 2.49)/ (15.73 ± 2.18)	(46.48 ± 1.99/ 21.05 ± 1.54)	(30.45 ± 2.43)/ (16.56 ± 2.66)	(70.25 ^a,b^ ± 3.01)/ (48.85 ^a,b^ ± 2.65)
HUVEC	(3.98 ± 1.56)/ (3.36 ± 3.20)	(6.66 ± 1.96/ 4.42 ± 2.41)	(5.58 ± 2.71)/ (3.92 ± 2.22)	(16.02 ^a^ ± 3.08)/ (7.64 ± 1.25)
HEK293	(18.7 ± 2.82)/ (7.66 ± 2.69)	(33.89 ^a^ ± 1.03)/ (13.48 ^a^ ± 1.65)	(36.68 ^a^ ± 1.05)/ (14.39 ^a^ ± 1.32)	(169.8 ^a,b^ ± 2.64)/ (54.33 ^a,b^ ± 2.47)

^a^ Significantly different from pure fucoxanthin treatment; ^b^ IC_50_ value in non-cancer cell significantly different from IC_50_ value in cancer cell.

Fucoxanthin is reported to have few adverse effects on normal cells both *in vitro* and *in vivo* [20,21]. At a single dose, mortality and abnormalities were found after dosing of 1000 and 2000 mg/kg in mice *in vivo* [22]. The 50% lethal dose of fucoxanthin was more than 2000 mg/kg body weight [10]. At repeated doses, oral administration of fucoxanthin (purity at 95%) at 750 mg/kg was applied to rats for 28 days did not show obvious toxicity [23]. Furthermore, histological study suggested no abnormality was found in different tissues including liver, kidney, spleen, and gonadal, after repeating dose of 500 and 1000 mg/kg for 30 day in mice [10]. In addition, the genotoxic/mutagenic effects were also not found in mouse bone marrow cells [24,25]. Thus, compared to existing cancer cytotoxic chemotherapy, fucoxanthin can generate growth inhibition effects to multiple types of cancer cell with low toxicity.

### 2.4. Growth Inhibition Effects of U. pinnatifida Extracts in Vitro

**Figure 4:**
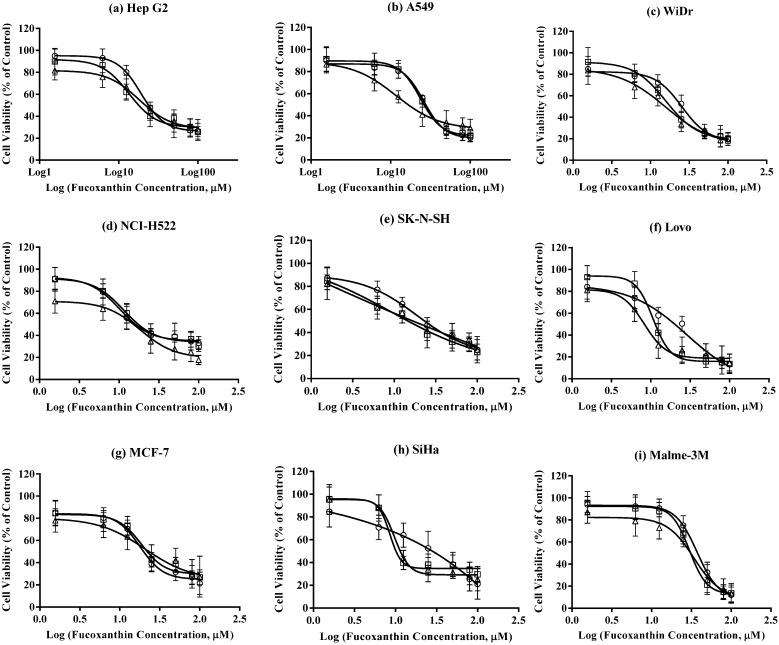
Inhibitory effects of three extracts from NZ *U. pinnatifida* (containing 0.2 (Δ), 43.5 (□) and 60.8% (○) fucoxanthin, respectively) on nine cancer cell lines. Data are expressed as mean ± standard errors (n = 6). (**a**) In Hep G2 cell line; (**b**) in A549 cell line; (**c**) in WiDr cell line; (**d**) in NCI-H522 cell line; (**e**) in SK-N-SH cell line; (**f**) in Lovo cell line; (**g**) in MCF-7 cell line; (**h**) in SiHa cell line; (**i**) in Malme-3M cell line.

All nine cancer cell lines were treated with 1.5625, 6.25, 12.5, 25, 50, 80, 100 μM of three *U. pinnatifida* extracts, including crude extract (containing 0.2% fucoxanthin), the first fucoxanthin extract (containing 43.5% fucoxanthin) and the second fucoxanthin extracts (containing 60.8% fucoxanthin) for 24, 48 and 72 h. Results are shown in Figure 4. Concentrations were normalized to the percentage of purity of fucoxanthin. 

Overall, the growth inhibition effects from all three NZ *U. pinnatifida* extracts fitted well with the Sigmoid model of negative exponential distribution, showing slightly decreased cell viability in the low concentration range followed by relatively steep drop of cell viability and then a plateau. Cancer cell viability significantly decreased with increased fucoxanthin concentration. 

IC_50_ values of pure fucoxanthin standard and our three extracts in cancer and non-cancer cell lines were compared in Table 2. After treatment with *U. pinnatifida* extracts for 48 and 72 h, significant differences in growth inhibition IC_50_ values between pure fucoxanthin and *U. pinnatifida* extracts were found in several cancer cell lines including WiDr, NCI-H522, SK-N-SH and Lovo (Table 2) (*p* < 0.05). The IC_50_ values of the crude NZ *U. pinnatifida* extract (0.2% fucoxanthin) for 48 h and 72 h were significantly lower than corresponding IC_50_ values of pure fucoxanthin standard. Especially for colon adenocarcinoma cell lines Lovo, the IC_50_ values of crude extract was almost three times lower than the IC_50_ values of pure fucoxanthin.

In human neuroblastoma cell line SK-N-SH, the IC_50_ values of the first and second extracts were also significantly lower than that of pure fucoxanthin (Table 2). Since the cells were exposed to the same concentration of fucoxanthin (normalized by purity in the experiment), this implicates that there may be a single unknown compound or a group of unknown compounds present in the NZ *U. pinnatifida* extracts that is effective in inhibiting cancer cell growth or can reduce cancer cell viability synergistically with the presence of fucoxanthin. We observed that the unidentified Peak C (in Figure 2) concentration decreased with further purification of fucoxanthin (increase in fucoxanthin purity of extracts), which also corresponded to the increase of IC_50_ value. Investigation of un-identified Peak C is currently underway. Another most interesting feature found in Table 2 is that IC_50_ values from crude extracts in non-cancer cell lines were significantly higher than the corresponding IC_50_ values from pure fucoxanthin treatment. Comparing IC_50_ values between cancer and non-cancer cell lines treated with crude extract, the IC_50_ values in two non-cancer lines were significantly higher than that in all nine cancer cell lines after 48 h and 72 h treatment. Furthermore, with the increase of fucoxanthin purity in extracts, the selectivity started to disappear; which indicates that the purification process eliminated the compound(s) that may have exerted its effect together with fucoxanthin. These findings clearly indicate that fucoxanthin with co-existing compounds in the crude extract can selectively inhibit cancer cell growth without exerting high toxicity to non-cancer cells. This provides a foundation for future functional food or biomaterial development from NZ *U. pinnatifida* extracts, especially in terms of cancer treatment or prevention. 

Most seaweed composition studies suggest that seaweeds offer a wide range of therapeutic possibilities, and report that seaweeds are low in fats but usually contain vitamins and bioactive compounds such as terpenoids and polyphenolic compounds [26,27,28]. In this study, the crude *U. pinnatifida* extracts were achieved by soaking its powder in pure methanol. In the process of extraction, pure hexane was used to remove non-polar substance such as carotene and chlorophyll compounds. Then silica gel chromatography was used as a purification process using normal phase chromatography. As a result, the extracts were removed from those known bioactive compounds. Therefore, the reason for the lower IC_50_ values of *U. pinnatifida* fucoxanthin extracts compared with fucoxanthin in brain tumor, colon, lung cancer cell lines should be the existence of currently un-identified (or novel) compound(s). Another compound that has growth inhibition effects in *U. pinnatifida* is fucoidan [4,5,7,8,29]. Fucoidan refers to a type of sulfated polysaccharide. The solubility of polysaccharide compound is reasonably high in water, and it has limited solubility in organic solvents such as methanol and ethanol [30,31]. Since we used organic solvent extraction, fucoidan was not present in the extracts in significant amount. This further strengthened our conclusion that novel cancer cell growth inhibition compound(s) exists in NZ *U. pinnatifida*. Such compound(s) exhibit selective toxicity against cancer cells either by themselves or by co-existing with fucoxanthin.

## 3. Experimental Section

### 3.1. Materials

#### 3.1.1. Materials for Fucoxanthin Extraction

Methanol, hexane, and chloroform were certified as HPLC grade and purchased from Thermofisher (Auckland, NZ). Silica gel powder (60–200 MESH) was purchased from J.T. Baker Chemical (Auckland, NZ), and benzene was from B.D.H. Ltd (Auckland, NZ). Pure fucoxanthin and internal standard canthaxanthin were purchased from Sigma-Aldrich (St. Louis, MO, USA).

#### 3.1.2. Materials for Cell Viability Assay

RPMI medium 1640, Cascade Biologic Medium 200, Dulbecco’s Modified Eagle Medium DMEM (1×), Low serum growth supplement (LSGS), L-glutamine-200mM (100×) liquid, Penicillin-Streptomycin (100 mL), Trypan Blue Stain and TrypLE™ Express Stable Trypsin Replacement Enzyme without phenol red were supplied by Invitrogen NZ Ltd (Auckland, NZ). Sterile filtered fetal bovine serum (FBS) was purchased from Medica Pacifica (Auckland, NZ). MTT [3-(4, 5-dimethylthiazol-2-yl)-2, 5-diphenyl tetrazolium bromide] was supplied by Sigma. DMSO (Dimethyl sulfoxide), was purchased by Merck-Chemicals (Poole, UK). 

#### 3.1.3. Human Cancer Cell Lines and Cell Culture

Human cancer cell lines used in this study were purchased from ATCC (Cryosite Ltd., Sydney, NSW, Australia), including lung carcinoma line A549, colon adenocarcinoma lines WiDr and Lovo, lung cancer line NCI-H522, hepatocellular carcinoma line Hep G2, neuroblastoma line SK-N-SH, breast adenocarcinoma line MCF, cervix squamous line SiHa, and malignant melanoma line Malme-3M. All nine cell lines were maintained in RPMI 1640 culture medium supplemented with 1% Penicillin-Streptomycin, 4 mM L-glutamine and 10% of FBS except Malme-3M (20% FBS). All cancer cell lines were maintained in tissue culture incubator at 37 °C and 5% carbon dioxide.

### 3.2. Human Non-Cancer Cell Lines and Cell Culture

Three human non-cancer cell lines including human dermal fibroblasts (HDFb), human umbilical vein endothelial (HUVEC) and human embryonic kidney (HEK293) were used to determine *in vitro* toxicity of pure fucoxanthin and extracts from NZ *U. pinnatifida*. All three cell lines were purchased from Invitrogen NZ Ltd. HDFb was maintained in Dulbecco’s Modified Eagle Medium supplemented with LSGS. HUVEC cells were maintained in Cascade Biologics Medium 200 supplemented with LSGS. HEK293 cells were maintained in RPMI 1640 culture medium supplemented with 1% Penicillin-Streptomycin, 4 mM L-glutamine and 10% of FBS.

### 3.3. Preparation of Fucoxanthin

#### 3.3.1. *U. pinnatifida* Sample Preparation

*U. pinnatifida* was collected from Port Underwood (Marine Farms PE327 and 106) and Pelorus Sound of Marlborough Sound (Marine Farms 122 and 353) in NZ, and the samples were collected at monthly intervals from June to December 2011. All sample preparation procedures have described in one of our previous publications [32].

#### 3.3.2. Fucoxanthin Extraction

*U. pinnatifida* samples were soaked in pure methanol and stirred mechanically for 48 h in dark condition at the room temperature to reduce the possibility of oxidation by sunlight. The ratio for extraction between *U. pinnatifida* sample and pure methanol is 100 g: 1 L. The extract was then filtered (Whatman No.1 filter paper, Thermofisher, Auckland, NZ) to remove the solids. This procedure was repeated until the seaweed powder was colorless. All methanol extracts were then combined. This was the crude *U. pinnatifida* extract. Hexane and distilled water were added into the crude fucoxanthin extract. The lower layer consisted of the methanol/water fraction was collected. The chloroform was then added into methanol/water extract. The ratio for pure methanol, pure hexane and pure chloroform was 15:15:10 (V/V/V). The mixture was then transferred to centrifuge for 15 min at room temperature, 12,000 rpm in a Sorvall RC5C instruments (Dupont, Wilmington, DE, USA) using the Fiberlite F21-8×50y rotor (Thermo-Fisher, San Jose, CA, USA). The organic layer was stored and dried at 30 °C using rotary evaporator. Then, the fucoxanthin fraction was freeze dried further to completely remove chloroform.

#### 3.3.3. Silica Gel Column Chromatography Purification

Due to a higher polarity solvent, pure benzene was used to dissolve the fucoxanthin sample. Pure hexane was the solvent in the silica gel column. There were four types of elution including pure hexane, about 300 mL of n-hexane/acetone (8:2, V/V) (which remove chlorophyll, carotenoids other than fucoxanthin and other impurities), about 400 mL of n-hexane/acetone (6:4, V/V) (which elute crude purified fucoxanthin out of the column) and acetone. 

Each fraction was collected and detected by using a UV-visible spectrophotometer (Ultrospec 2100, UV-visible Amersham Pharmacia Biotech, Cambridge, UK) at 450 nm. Fractions that had high absorbance values at 450 nm were combined. This was the first fucoxanthin extract. Half of the extract was dried using a rotary evaporator at 30 °C and freeze-dried in a freeze dryer overnight. The remaining half of the first fucoxanthin extract was put back into the silica column repeating the procedure above to obtain the second fucoxanthin extract. The second fucoxanthin extract was dried using a rotary evaporator and sample was freeze dried in a freeze dryer overnight. Before drying two fucoxanthin extracts above, the purified fucoxanthin was quantified using HPLC. Both first and second fucoxanthin extract were flushed with argon gas and stored in −80 °C freezer until further use.

#### 3.3.4. HPLC Analysis

Fucoxanthin was determined using a HPLC method [33]. HPLC system consists of a LC-20AT pump system (Shimadzu, Tokyo, Japan), a UV-Vis SPD-20A (Shimadzu) absorbance detector and online analysis software (LC Solution version 1.25). The separation was achieved by using a Luna 5 μM C18 (4.6 mm × 250 mm, Phenomenex, Torrance, CA, USA) reverse phase column. The mobile phase was HPLC graded pure methanol at flow rate of 1 mL/min. The volume of sample injection was 50 μL. The wavelength for fucoxanthin detection was set at 450 nm.

Pure fucoxanthin with concentration range of 0.0078–0.125 μg/mL in pure methanol was used to construct a standard calibration curve. Canthaxanthin standard (0.0625 μg/mL) was dissolved in pure acetone and used as an internal standard. This HPLC method has been validated as described in our previous publication [32].

### 3.4. Cell Viability Assay

Cell viability was determined by MTT assay as previously described [34] with a slight modification. Briefly, the optimized seeding cell density was 5000 cells/well for A549 and WiDr, while for Hep G2, Malme-3M, SiHa, NCI-H522, Lovo, SK-N-SH, and MCF-7, the seeding cell density was 10,000 cells/well. Microplate with 96 wells was used for the experiment and each well contained 100 μL of medium with cancer cells. Treatment time was 24, 48 or 72 h. All cancer cell lines were cultured in the presence or absence of fucoxanthin extract or standard. 

Pure fucoxanthin standard and all *U. pinnatifida* fucoxanthin extracts were dissolved in pure ethanol to 5 mM as a stock solution, and the final ethanol concentration was 2% in the culture medium. An aliquot of 20 μL of MTT reagent in phosphate buffer saline at a concentration of 5 mg/mL was added into each well after experiment, and washed with 150 µL of PBS. After incubation for 4 h at 37 °C, the formed MTT-formazan was dissolved in 150 μL DMSO. The viable cell amount was determined by the absorbance at 540 nm using a multiplate-reader (UV-visible FLUOStar Omega Multidetection Microplate Reader, AlphaTech NZ Ltd., Auckland, New Zealand). The IC_50_ calculated represents the effective concentration of extract which gives 50% inhibition of the cell growth and the IC_50_ value was generated by using Prism (GraphPad, La Jolla, San Diego, CA, USA) software.

### 3.5. Statistical Analysis

All results were examined by statistic software Minitab^®^ (Version 16). Two-way ANOVA Turkey’s multiple comparison tests was carried out. Compared with control group (the group without standard or extract treatment added), the cancer cell growth inhibition effects of treatment was considered to be significant if the *p* value was <0.05. To compare treatments, one way ANOVA with Turkey’s test was employed to compare crude *U. pinnatifida* extract, first fucoxanthin extract and second fucoxanthin extract. 

## 4. Conclusions

NZ *U. pinnatifida* contains fucoxanthin, which possesses growth inhibition effects on multiple types of cancer cells, in particular melanoma and cervix squamous carcinoma. At a low concentration, fucoxanthin showed selective cytotoxicity to one malignant melanoma line. Therefore, NZ *U. pinnatifida* could be a potential source of fucoxanthin for pharmaceutical use to treat or prevent those two types of cancer, or could be used as a type of food for health to reduce the incidence of those two types of cancer. It is also possible that fucoxanthin could be used at relative low doses in the combination chemotherapy with other cytotoxic anticancer drugs, because at low concentration, it exhibits selective cancer cell growth inhibition effect to cancer cells. The limitation of the current study is that *in vivo* conditions are always different from *in vitro* conditions, and our findings may not be able to be translated into *in vivo* success. A thorough mechanism of action study would have provided more clues for potential future applications of fucoxanthin. 

This is the first study to investigate extracts from NZ *U. pinnatifida*, which has not been comprehensively studied before because of its invasive species status in the past. It is highly likely that NZ *U. pinnatifida* extracts contain novel compound(s) with selective cancer cell growth inhibition effects in cancer cells, especially in human colon adenocarcinoma, lung carcinoma and neuroblastoma. Co-existence of fucoxanthin and the unknown compound(s) in NZ *U. pinnatifida* extract exhibits selective toxicity against cancer cells, but not to non-cancer cells. Additional research is needed to identify such compound(s), which would be highly desirable in the field of cancer research. Our research has provided the foundation for such further investigation. Nevertheless, even without identifying the actually compound(s), it can be concluded that NZ *U. pinnatifida* is a resource rather than a marine pest. It can definitely be used as a source for either functional biomaterial extraction or functional food production. 
